# Respiratory Syncytial Virus (RSV) Hospitalizations in the Elderly in a Tertiary Care Hospital in Southern Italy as a Useful Proxy for Targeting Vaccine Preventive Strategies

**DOI:** 10.3390/idr16030037

**Published:** 2024-05-31

**Authors:** Francesca Centrone, Daniela Loconsole, Alfredo Marziani, Valentina Annachiara Orlando, Arianna delle Fontane, Martina Minelli, Maria Chironna

**Affiliations:** 1 Hygiene Unit, Azienda Ospedaliero-Universitaria Consorziale Policlinico di Bari, 70124 Bari, Italy; francesca.centrone.fc@gmail.com; 2 Hygiene Section, Department of Interdisciplinary Medicine, University of Bari, 70124 Bari, Italy; daniela.loconsole@uniba.it (D.L.); alfredomarziani.md@gmail.com (A.M.); v.orlando11@studenti.uniba.it (V.A.O.); ariannadellefontane@gmail.com (A.d.F.); dr.martinaminelli@gmail.com (M.M.)

**Keywords:** RSV infection, viral infectious diseases, elderly, vaccination, RSV vaccine, hospitalization, Southern Italy, prevention strategy, surveillance

## Abstract

RSV infection causes severe respiratory illness and mortality in the elderly, especially in the presence of comorbidities. Early identification of infection would result in appropriate clinical-therapeutic management, avoiding hospitalizations, the risk of healthcare-associated infections, and inappropriate antibiotic prescriptions, thus reducing healthcare costs and fighting antimicrobial resistance. The aim of this study was to assess RSV hospitalizations in subjects >64 years hospitalized in a large tertiary care hospital in Southern Italy, in order to assess their usefulness as a proxy for targeting a potential vaccination strategy. Fifty-two RSV-positive patients were identified from the 2014–2015 to the 2022–2023 seasons. RSV type B was found in 71.2% of cases. The median age was 78 years (IQR: 72–84) and 40.4% of the subjects had at least one comorbidity; 5.8% needed intensive care. The use of combined rapid tests for SARS-CoV-2/influenza/RSV identification in primary care settings may contribute to an improved definition of the burden of RSV in the elderly. The implementation of an anti-RSV vaccination strategy in the elderly population would reduce direct and indirect infection costs. More robust epidemiological data in Italy are needed for targeted preventive strategies.

## 1. Introduction

Respiratory Syncytial Virus (RSV) is an RNA virus belonging to the Pneumoviridae family, Orthopneumovirus genus, recognized as one of the major pathogens responsible for respiratory illnesses and mortality in the pediatric population, especially in the first six months of life. In 2019 alone, globally, 33 million RSV infections, 3–6 million hospitalizations due to RSV infection, and more than 26 thousand deaths due to RSV infection in children aged 0–60 months were reported [1]. RSV is not a cytopathic virus and replicates almost exclusively in the apical ciliated cells of the respiratory tract mucosa, mainly causing superficial damage. This damage predisposes the patient to secondary bacterial superinfections [2]. Over the last few decades, RSV has also been recognized as one of the most common causes of acute respiratory infections in adults [2].

RSV is an extremely contagious virus that can cause respiratory infections both in community and hospital settings, with a transmission risk ranging from 6–12% in hospital units with immunocompromised adults to 30–32% in other adult wards [3]. Transmission occurs by direct and indirect airborne routes, through contact of the mucous membranes of the eyes, nose and mouth with droplets containing the virus [2].

RSV follows a seasonal pattern, and outbreaks show a two-yearly oscillation model, probably driven by ecological factors and short-lived specific immunity that, based on mathematical models, is estimated to last 6–12 months [4]. In the temperate climate regions of the northern hemisphere, RSV infections peak during the fall–winter season, usually between December and January, but may continue to occur until late spring. There are two subtypes of RSV, A and B, which alternate each year, and one of the two subtypes generally predominates during a single season, also depending on regional variations [2].

Most cases of RSV disease in adults occur in the elderly, and in the United States, an estimated 60,000 to 160,000 hospitalizations and 6000 to 10,000 deaths occur annually in individuals aged 65 years and older [5]. Despite the limited data available in the adult population, RSV in Italy is estimated to be responsible for approximately 290,000 cases of acute respiratory infection (ARI), 26,000 hospitalizations, and 1800 deaths each year in individuals over 60 years of age [6].

Adults with RSV infection may manifest non-specific symptoms, predominantly presenting with influenza-like illness (ILI), nasal congestion, rhinorrhea and sore throat [2]. In the elderly, however, RSV infection can lead to severe forms characterized by pneumonia and respiratory failure, often resulting in hospitalization and death, mainly in the presence of comorbidities such as bronchopneumonia, chronic obstructive pulmonary disease (COPD), asthma, cardiac failure, diabetes and immunodeficiency [2]. Moreover, compared to influenza, recent studies suggest that RSV may be associated with more severe outcomes, including longer hospital stays, increased incidence of pneumonia, and higher rates of admission to intensive care units and one-year mortality following hospitalization [7]. In Italy, a report on RSV infection in adults over 65 years of age showed a hospitalization rate of 93.2% [8]. Among those aged over 50, the average length of hospital stay for RSV infection is 3–6 days, with an overall mortality rate of 6–8% [9]. For this age group, it has been estimated that 10–31% of patients require admission to intensive care and 3–17% require mechanical ventilation [9]. Available epidemiological studies mainly focus on the burden of hospitalizations and RSV infections in the pediatric population, while studies on the impact of RSV on adults remain limited. This is due to several issues, such as the lack of a uniform definition of cases, the heterogeneity of tests used for diagnosis, and the low aptitude for laboratory diagnosis [10]. Additionally, few studies are available on the burden in community settings [10].

To date, although there is no systematic surveillance of RSV infections in Western countries, some efforts have been made to improve the monitoring of RSV infections. In particular, the WHO piloted a Global Respiratory Syncytial Virus Surveillance program based on the Global Influenza Surveillance and Response System and, in Europe, the European RSV Surveillance Bulletin—PROMISE and the RESCEU project were launched [11,12,13]. In Italy, in the 2022–2023 season, integrated surveillance of respiratory viruses (RespiVirNet) was set up based on the reporting of ILIs observed by sentinel physicians (general practitioners and community pediatricians) and the monitoring of the circulation of influenza and respiratory viruses, including RSV [14].

Currently, there are no specific therapies for the treatment of RSV infection in adults. Therefore, it is crucial to focus on the implementation of prevention strategies. In Italy, since October 2023, two anti-RSV vaccines have been licensed for the prevention of lower respiratory tract infections (LRTIs), both recommended for the immunization of individuals aged 60 years and older [15]. However, to date, a vaccine strategy has not yet been defined to limit the impact of RSV infections.

The aim of this study was to assess and describe RSV hospitalizations in subjects older than 64 years hospitalized in a large tertiary care hospital in Southern Italy, in order to assess their usefulness as a proxy for delineating a potential vaccination strategy.

## 2. Materials and Methods

A retrospective observational study was conducted on subjects aged over 64 years old, hospitalized at the A.O.U.C. Policlinico in Bari with ARI, with a request for investigation for RSV infection and confirmed as positive for RSV infection, in the seasons from 2014–2015 to 2022–2023. The hospital is one of the most important teaching hospitals in Southern Italy, serving a population of approximately 350,000 inhabitants. Diagnosis was performed through real-time PCR on nasopharyngeal swabs collected from patients with symptoms of ARI. Samples were collected in the hospital wards and sent to the Laboratory of Molecular Epidemiology and Public Health of the U.O.C. Igiene—A.O.U.C. Policlinico in Bari, where they were processed immediately or frozen at −80 °C until testing. Nucleic acids were extracted using the STARMag Universal Cartridge kit on the automated Nimbus IV platform (Seegene, Seoul, Republic of Korea). For diagnosis, a commercial real-time PCR was used (AllplexTM Respiratory Panel Assays, Seegene, Seoul, Republic of Korea) for the detection of 16 viruses: influenza A and B, RSV A and B, adenovirus, enterovirus, parainfluenza viruses 1–4, metapneumovirus, bocavirus, rhinovirus and human coronaviruses NL63, 229E and OC43. From 2020, the samples were also tested for SARS-CoV-2 using the implemented commercial test (AllplexTM Respiratory Panel Assays, Seegene, Republic of Korea).

For the purpose of this study, each season was between week 42 of the year and week 41 of the following year according to the Italian respiratory virus surveillance, and the peak of RSV infections was identified as the month with the highest number of infections recorded in the season. For each enrolled subject, demographic characteristics, comorbidities and coinfections were collected retrospectively. Statistical analysis was performed using StataMP12.0^®^ (StataCorp LLC, College Station, TX 77845-4512, USA).

## 3. Results

**Figure 1:**
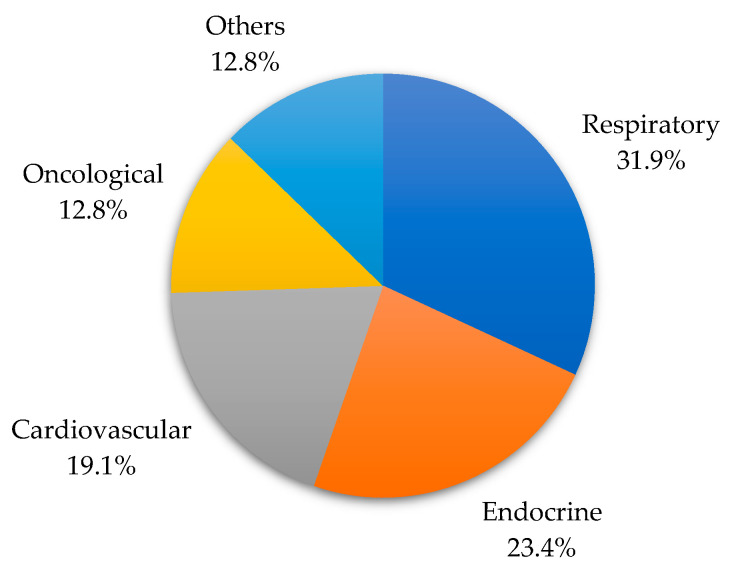
Distribution of elderly patients with RSV infection by comorbidities.

A total of 52 patients over 64 years of age with RSV infection were identified during the study period. RSV type A was detected in 28.8% of cases and RSV type B in 71.2%. Among the enrolled subjects, 59.6% were male. The median age was 78 years (interquartile range, IQR: 72–84), and 63.5% were older than 74 years. One or more comorbidities were present in 40.4% of the patients. The distribution of patients by comorbidities is shown in Figure 1.

In Table 1, the characteristics and comorbidities of the study population are reported.

**Figure 2:**
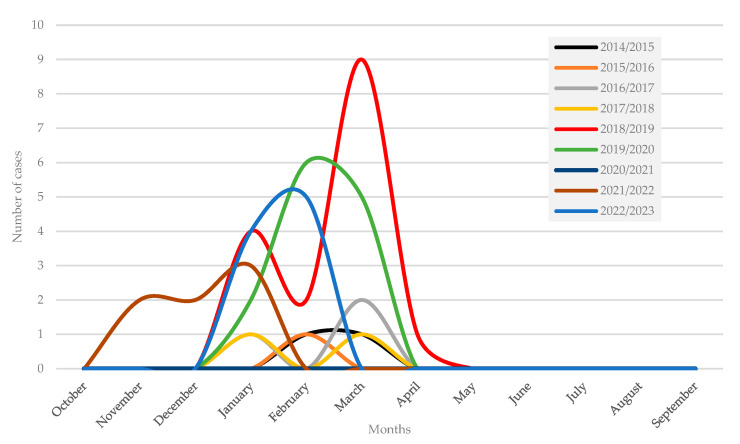
Distribution of RSV infections in hospitalized patients by month of diagnosis, seasons 2014/2015–2022/2023.

Of the enrolled patients, 30.8% were hospitalized in internal medicine, 15.4% in pulmonology, 15.4% in the emergency department, 11.5% in infectious diseases, 5.8% in intensive care unit (ICU) and 21.2% in other medical wards. Seven patients (13.5%) had coinfection with another virus, specifically three with rhinovirus, two with coronavirus OC43, one with bocavirus and one with SARS-CoV-2. Among the patients admitted to the ICU, one patient was 67 years old, had no comorbidities and had an RSV B/rhinovirus infection. The other two were older than 80 years; both had no comorbidities and had an RSV A infection. Figure 2 shows the distribution of RSV hospitalized cases by month of diagnosis and seasons.

In the 2018–2019 season, 30.8% of cases (*n* = 16) were recorded. In the 2020–2021 season, no cases of infection were recorded due to the COVID-19 pandemic. In pre-pandemic seasons, the peak of hospitalizations for RSV infection was recorded between February and March. However, in the 2021–2022 and 2022–2023 seasons, the peak of RSV epidemics was recorded earlier than in previous seasons, in January and February, respectively. In addition, in the 2021–2022 season, compared with the other seasons analyzed, cases were recorded from November 2021. Among the post-pandemic seasons, the highest number of cases was recorded in 2022–2023.

## 4. Discussion

While the impact of RSV on children in terms of hospitalizations and community burden is gradually becoming more defined, the impact of RSV infections on adults and the elderly is underestimated due to limited available data. Planning preventive strategies to reduce the burden of this infection, especially in individuals older than 65 years, requires more detailed studies in this age group. Currently, the few available studies are based on the recruitment of patients with symptoms of ILI, ARI or SARI (severe acute respiratory infection), and most surveillance systems already in place also rely on the detection of symptomatic infections (e.g., ILIs). Adults with RSV infection have a strong cellular immunological memory and thus are often asymptomatic or have mild symptoms, but can be an important reservoir of infection [4]. Therefore, more appropriate patient recruitment methods are needed to estimate the real RSV burden in adults, as the seasonality and clinical manifestations of RSV infection may differ from those of influenza virus infection. In addition, a reduced aptitude for laboratory diagnosis has been observed in adult patients with ARI [16].

Data from this study show that, during the period analyzed, RSV type B was detected more frequently in hospitalized subjects older than 65 years than type A, with a ratio of about 2:1. This finding appears to be in contrast to that observed in a study by Shi T. et al. who reported a higher risk of hospitalization in subjects in the same age group with type A infection [17]. However, this discrepancy could be attributed to the different sample sizes of the studies. The median age of patients recruited during the period analyzed was 78 years and, of note, the majority of hospitalized elderly subjects (63.5%) were older than 74 years. These data are consistent with the results reported in the systematic review by Cong B et al. who observed a higher rate of RSV hospitalizations in the over-75s, second only to children under 4 years of age [18].

Among the patients enrolled, approximately 40% had underlying comorbidities. As reported in the literature, adults at higher risk of RSV hospitalization are those affected by previous cardiovascular and respiratory diseases [19]. A recent study conducted in Germany, which examined RSV hospitalizations from 2010 to 2019, showed chronic lower respiratory tract disease in 24% of adult patients [20]. Meanwhile, in our study focused on elderly hospitalized patients with RSV infection, 32.0% showed respiratory comorbidities. Viral coinfection was detected in 13.5% of the patients studied. It is known that patients with viral coinfection are at higher risk of hospitalization and worse outcomes [21]. Furthermore, some studies identify RSV infection as an independent risk factor for hospitalization and intensive care demand [22]. Most of this evidence derives from studies in the pediatric population. In adults and the elderly, however, it is bacterial coinfection, rather than viral, that contributes most to the unfavorable evolution of the disease [23]. Nevertheless, it is worth mentioning that all patients in the present study who were admitted to the ICU had no comorbidities.

It has been shown that the pandemic altered the typical patterns of RSV in temperate areas [24]. In the pre-pandemic seasons analyzed in this study, the highest circulation of RSV was recorded in the winter period, a finding consistent with previously reported data [25,26]. The absence of cases in the 2020–2021 season was observed in Italy and also in other countries, including the United States, and could be partly explained by the public health measures introduced during the COVID-19 pandemic, in particular, non-pharmacological prevention interventions (NPIs) such as masks and social distancing [25,26]. A subsequent increase in pediatric RSV cases, higher than the seasonal average peak in pre-pandemic years, was first detected in the southern hemisphere and then confirmed in the northern hemisphere as well [26]. In the present study, the seasonal peak of infections in the elderly followed that of the pediatric population. This change in RSV epidemiology could be attributed to both the relaxation of NPIs and the presence of more susceptible individuals in the population. The epidemic peak of RSV infections in the 2022–2023 season, however, occurred in February–March. Although these data suggest that seasonal RSV trends are likely returning to pre-pandemic levels, as observed in other countries, no data are currently available to definitively support this change [25].

Despite the limited data available on RSV infection in the elderly population in Italy, the present study has some limitations. Firstly, the data were obtained from the database of admissions in only one hospital. Secondly, the sample size is small and could be under-represented by the lack of laboratory diagnosis even in the hospital setting. Furthermore, regarding coinfections, we cannot differentiate them from viral shedding. Therefore, caution is needed when interpreting the data. Further studies on a larger population of elderly people are needed.

## 5. Conclusions

The burden of RSV infections in the adult and elderly population is difficult to estimate due to the lack of a specific surveillance system and the low tendency to diagnosis, especially in this age group. Therefore, it is essential to set up systematic surveillance of RSV infection cases in the elderly population in order to obtain robust epidemiological data to plan appropriate preventive strategies. In this perspective, the introduction of combined rapid tests for the identification of SARS-CoV-2/influenza/RSV in primary care could help to improve the estimation of the burden of RSV in the elderly. Thus, diagnoses made by general practitioners (GPs) could be combined with virological surveillance of influenza, which is already in place, and contribute to year-round data collection. Furthermore, early identification of the infection would lead to appropriate clinical–therapeutic management. Correct diagnosis would avoid inappropriate antibiotic prescriptions, which would reduce healthcare costs and slow antimicrobial resistance. In addition, timely diagnosis could lead to improved outcomes for these patients, avoiding hospitalizations and reducing the risk of healthcare-associated infections. Considering the aging of the population and the increasing presence of comorbidities, the implementation of a vaccination strategy against RSV in the elderly population would reduce the direct and indirect costs of infections in the long term.

The availability of more robust epidemiological data is crucial for targeting appropriate preventive strategies. The Italian Ministry of Health and subsequently the Italian Society of Hygiene and Preventive Medicine have recommended the introduction of anti-RSV vaccination in individuals aged 75 years or older and in those aged 60 years or older with comorbidities. Since the majority of hospitalized patients with RSV infection were subjects over 74 years of age, these interim recommendations seem to be in line with the results of our study.

## Figures and Tables

**Table 1:** Characteristics and comorbidities of the study population.

		N	%
Total		52	100.0%
Sex	Female	21	40.4%
	Male	31	59.6%
Age, years	65–70	9	17.3%
	71–74	10	19.2%
	75–79	10	19.2%
	>80	23	44.3%
Comorbidities		21	40.4%
	Respiratory	15	28.8%
	Cardiovascular	9	17.3%
	Diabetes	11	21.2%
	Oncologic	6	11.5%
	Others	5	9.6%
Coinfections		7	13.5%

## Data Availability

The data presented in this study are available on request from the corresponding author due to privacy concerns.
